# Altered Inhibitory Control and Increased Sensitivity to Cross-Modal Interference in Tinnitus during Auditory and Visual Tasks

**DOI:** 10.1371/journal.pone.0120387

**Published:** 2015-03-12

**Authors:** Rodrigo Araneda, Anne G. De Volder, Naïma Deggouj, Laurent Renier

**Affiliations:** 1 Institute of Neuroscience (IoNS), Université catholique de Louvain, Brussels, Belgium; 2 Department of Oto-Rhino-Laryngology & Head and Neck Surgery, Université catholique de Louvain and clinique universitaire St-Luc, Brussels, Belgium; University of Salamanca- Institute for Neuroscience of Castille and Leon and Medical School, SPAIN

## Abstract

Tinnitus is the perception of sound in the absence of external stimulus. Currently, the pathophysiology of tinnitus is not fully understood, but recent studies indicate that alterations in the brain involve non-auditory areas, including the prefrontal cortex. In experiment 1, we used a go/no-go paradigm to evaluate the target detection speed and the inhibitory control in tinnitus participants (TP) and control subjects (CS), both in unimodal and bimodal conditions in the auditory and visual modalities. We also tested whether the sound frequency used for target and distractors affected the performance. We observed that TP were slower and made more false alarms than CS in all unimodal auditory conditions. TP were also slower than CS in the bimodal conditions. In addition, when comparing the response times in bimodal and auditory unimodal conditions, the expected gain in bimodal conditions was present in CS, but not in TP when tinnitus-matched frequency sounds were used as targets. In experiment 2, we tested the sensitivity to cross-modal interference in TP during auditory and visual go/no-go tasks where each stimulus was preceded by an irrelevant pre-stimulus in the untested modality (e.g. high frequency auditory pre-stimulus in visual no/no-go condition). We observed that TP had longer response times than CS and made more false alarms in all conditions. In addition, the highest false alarm rate occurred in TP when tinnitus-matched/high frequency sounds were used as pre-stimulus. We conclude that the inhibitory control is altered in TP and that TP are abnormally sensitive to cross-modal interference, reflecting difficulties to ignore irrelevant stimuli. The fact that the strongest interference effect was caused by tinnitus-like auditory stimulation is consistent with the hypothesis according to which such stimulations generate emotional responses that affect cognitive processing in TP. We postulate that executive functions deficits play a key-role in the perception and maintenance of tinnitus.

## Introduction

Tinnitus is the perception of sound in the absence of a corresponding external acoustic stimulus. Its prevalence has increased in the recent years, affecting up to 20% of the general population in western countries, while it severely impairs the quality of life of about 1–2% of all people [1–2]. Despite its high prevalence and the increasing interest from the scientific community, the pathophysiology of tinnitus is not fully understood yet. For long, tinnitus was thought to be a strict otological disorder, but advances in neuroimaging methods and the development of animal models have shifted the perspective towards the neural correlates underlying tinnitus [3–8]. Today, accumulating evidence points towards a role of non-auditory brain areas in tinnitus, including the prefrontal cortex [7, 9–13]. In particular, transcranial magnetic stimulation (TMS) and transcranial direct current stimulation (tDCS) studies revealed a dysregulation of ventrolateral and dorsolateral prefrontal cortex in tinnitus [8, 14], which are two brain regions associated with inhibitory control (i.e. the ability to suppress inappropriate behavioral responses and often conceptualized as an executive function) [15–17]. Currently, very few studies have investigated the executive functions in tinnitus patients (e.g. using Stroop tasks), and the importance and the nature of the cognitive deficits accompanying tinnitus are not totally clear yet [18–23].

Here, we hypothesize that altered executive functions in tinnitus are causing difficulties to ignore or inhibit irrelevant stimulations which would lead to both phantom perceptions (tinnitus) and an increased sensitivity to sensory interference (e.g. distractibility). This hypothesis is in line with recent models that postulate that the habituation process is hampered in tinnitus patients [22, 24] and that the prefrontal cortex plays a role in the so-called noise cancelling pathway [4, 7] and/or in the conscious perception of tinnitus [6, 8].

The purpose of the present study was to test in a go/no-go paradigm in the visual and auditory modalities to what extent tinnitus participants (TP) have impaired inhibitory control and are more sensitive to sensory interference than matched control subjects (CS).

## Methods

### Ethics Statement

All participants provided their written informed consent prior to the study according to the Declaration of Helsinki (BMJ 1991; 302: 1194). The experimental protocol of the study was approved by the Biomedical Ethics Committee of the school of Medicine of the Université catholique de Louvain.

### Participants

Tinnitus participants (TP) were recruited among patients who consulted the ear, nose and throat (ENT) department of the St-Luc academic hospital in reason of tinnitus. Strict inclusion criteria were used in order to homogenize the best possible the group of tinnitus patients since there may be different types of tinnitus baring different characteristics and involving slightly different pathophysiological mechanisms [2, 5, 25–26]. A special attention was paid to isolate the tinnitus from potential confounding factors that are frequently associated to tinnitus such as hearing loss, depression, anxiety or hyperacusis [27–28]. Therefore, we only included patients who suffered from a subjective tinnitus (1), non-pulsatile (2), permanently present (3), for at least 6 months (chronic) (4), in both ears (5), with at least one sound-frequency that was clearly identifiable (6), who had either a normal hearing acuity or a slight (sensorineural) hearing loss (i.e. average hearing loss for tonal stimuli inferior to 35 dB in each ear) (7), no hyperacusis (8), no neurologic record or diagnosed psychiatric disorder (including major depression) at the time of the testing (9), and no psychotropic medication consumption (10). In total, 17 tinnitus patients (TP) accepted to participate and were included in the study (6 men, 11 women, mean age: 49.0, SD: 15.2, ranging from 20 to 67 years; see Table 1 for details). Control subjects (CS) were recruited via flyers posted on the university campus and at St-Luc hospital. All of them were healthy, without self-reported neurologic or psychiatric problem or hearing impairment. All TP and CS were French speakers and underwent a brief audiometric evaluation of each separate ear (for 250, 500, 1000, 2000, 3000, 4000, 6000 and 8000 Hz frequencies) using a staircase ascending method (bottom-up approach). In addition, the tinnitus frequency was identified in each patient by presenting pure tones and narrow band sounds of frequencies ranging between 250 and 8000 Hz. Subjects had to determine which type of sound, frequency and intensity was the closest to their tinnitus perception. Seventeen CS were selected and individually matched to a TP for gender, age (CS: 48.8 ± 14.6), hearing acuity (each subject being categorized as having either (1) a normal hearing (i.e. hearing loss (hl) <20dB), or (2) slight hearing loss (hl between 21 and 35 dB (see Table 1)) and educational level (amount of education years; CS: 15.2 years ± 2.1). Fourteen subjects were right-handed in each group (3 left-handed).

**Table 1:** Characteristics of Tinnitus participants.

Subjects	Age [years]	Gender	Hearing Loss Right/Left [dB]	Tinnitus Frequency [Hz]	Tinnitus Duration [months]	THI [Score: 0–100]	Tinnitus Annoyance [VAS score: 0–10]
1	65	F	31/32	4000	72	24	7
2	54	F	11/12	8000	60	82	8
3	23	F	15/16	1500	33	46	2
4	62	F	23/20	4000	74	82	6
5	68	M	24/25	4000	24	56	4
6	29	F	2/7	1500	10	22	0
7	36	F	17/14	6000	15	76	5
8	44	F	11/10	6000	12	44	8
9	67	M	15/17	8000	24	48	5
10	60	M	12/8	8000	8	44	3
11	36	M	11/14	6000	11	34	0
12	56	F	17/14	6000	240	28	2
13	54	M	5/7	6000	13	24	1
14	20	F	9/11	6000	24	40	10
15	48	M	12/17	4000	9	32	4
16	58	F	18/14	6000	164	24	2
17	53	F	23/20	8000	7	30	7

THI: Tinnitus Handicap Inventory.

VAS: Visual Analogue Scale.

Hearing loss: Averaged scores (250–8000 Hz)

### Questionnaires

All TP filled out five questionnaires or scales recommended by the Tinnitus Research Innitiative (TRI) (http://www.tinnitusresearch.org/index.php) and/or commonly used by clinicians and researchers in the field: the Tinnitus Sample Case History Questionnaire (TSCHQ), the Tinnitus Handicap Inventory (THI), the Beck Depression Inventory (BDI), the Self-Rating Depression Scale (SDS) and the Beck Anxiety Inventory (BAI). The TSCHQ allows identifying the tinnitus history and characteristics [27] while the THI provides an evaluation (between 0 and 100) of its impact on daily living [29]. The depression and anxiety scales were used to control the potential effect of emotional state on performance to our tests. In addition, the annoyance caused by tinnitus was assessed on the day of the testing using a visual analogue scale (VAS); each participant had to rate the level of subjective discomfort in link with his tinnitus by marking a 10 cm line whose left extremity represented the lowest degree of discomfort (no discomfort). Table 2 shows the scores to the audiometry, questionnaires and scales in TP and CS.

**Table 2:** Clinical evaluation and scores to the questionnaires.

	Tinnitus (n = 17)	Control (n = 17)	unpaired t-test
	Mean (SD)	Mean (SD)	
Age [years] ^**ns**^	49.00 (15.22)	48.76 (14.63)	p = 0.4818
Bilateral Hearing Loss [db] ^**ns**^	15.17 (7.26)	12.21 (6.86)	p = 0.0953
Educational level [years] ^**ns**^	14.41 (3.04)	15.24 (2.05)	p = 0.1807
Beck Depression Inventory [13 items] ^**ns**^	2.94 (2.75)	2.41 (2.96)	p = 0.5404
Zung Self-Rating Depression Scale ^**ns**^	0.47 (0.09)	0.44 (0.10)	p = 0.1966
Beck Anxiety Inventory ^**ns**^	6.82 (2.19)	5.47 (2.62)	p = 0.0761

Bilateral Hearing Loss: Average between 250–8000 Hz

## Experiment 1

In this first experiment, we used a go/no-go paradigm to evaluate the inhibitory control in tinnitus participants (TP) and matched control subjects (CS) in unimodal (auditory, visual) as well as in bimodal (auditory-visual) conditions. The effect of the sound frequency used for the targets and the distractors on performance was also tested by comparing tinnitus-matched/high frequency versus low frequency sounds in the auditory conditions.

In addition, a stimulus detection task was used to measure the reaction speed in response to simple stimulations in the auditory and the visual modalities. In these conditions, the stimulus processing was kept to a minimum and little attentional resources were required. Therefore, these conditions were thought to represent the lowest level of sensory processing.

### Material and Stimuli

The auditory stimuli consisted of two pure tones: one fixed low frequency (440 Hz) and one high frequency that was individually matched to the tinnitus perception of each patient. The auditory stimuli were presented via headphones (HD 280 Sennheiser Pro). The intensity of the auditory stimulation was individually adapted based on the hearing profile of each subject; each sound was delivered 35 dB above the hearing threshold of each participant. The stimulus duration was 250 ms with a rise and fall time of 10 ms. During the auditory conditions, the subjects were gazing at a fixation cross displayed on a computer screen. Visual stimuli consisted in two grey circles: one small (3 mm radius) and one large circle (7 mm radius). They were displayed at the centre of a computer screen on a black background. Between each stimulus presentation a pale grey fixation cross was displayed in the same location as the circle. In all the conditions the stimulus presentation sequence was randomized. There were 72 trials in each condition and each stimulus was presented for 250 ms with a variable inter-stimulus interval (ISI) of 450, 650 or 850 ms. In the go/no-go conditions, targets (go) were presented in 80% of the trials and the distractors (no-go) in 20%. Stimulations were controlled and subjects’ responses were recorded using Matlab R2007b (The MathWorks Inc., Natick, MA, 1984–2007). Participants delivered their responses by pressing a button using a mouse of high temporal accuracy (Razer, model number: RZ01–0015).

### Experimental conditions

Target detection speed (Stimulus detection) was evaluated in one visual and two auditory conditions. There were five go/no-go conditions: two unimodal auditory, one unimodal visual and two bimodal auditory-visual conditions. Each auditory condition (stimulus detection, unimodal go/no-go and bimodal go/no-go) comprised two sub-conditions (using either a tinnitus-matched/high frequency or a low frequency sound) to assess the effect of the sound frequency on performance.

### Tasks and Procedures

**Fig 1:**
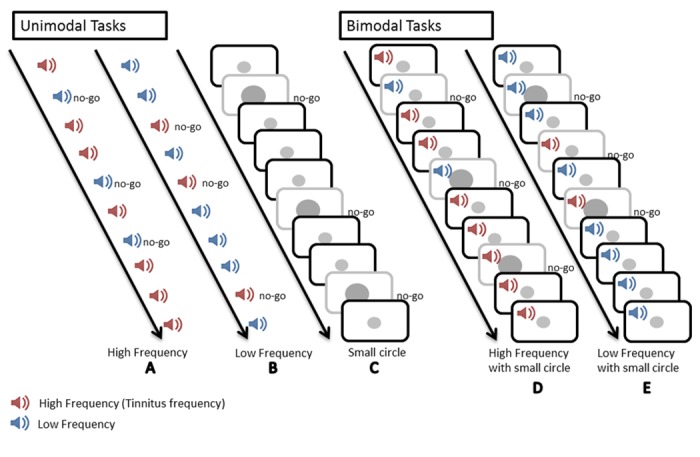
Illustration of the go/no-go conditions. (A) An unimodal auditory task. Participants were instructed to press a mouse button at the presentation of the target (tinnitus/high frequency sound (in red)) and not at the presentation of the distractor (a low frequency sound (in blue)). (B) An unimodal auditory task. Participants were instructed to press a mouse button at the presentation of the target (a low frequency sound (in blue)) and not at the presentation of the distractor (a tinnitus/high frequency sound (in red)). (C) A unimodal visual task. Participants were instructed to press a mouse button at the presentation of the target (the small circle) and not at the presentation of the larger circle (distractor). (D) A bimodal condition. Participants were instructed to press a mouse button at the presentation of the target (combination of the small circle and the tinnitus/high frequency sound) and not at the presentation of the distractors (the other combinations). (E) A bimodal condition. Participants were instructed to press a mouse button at the presentation of the target (combination of the small circle and the low frequency sound) and not at the presentation of the distractors (the other combinations).

The testing took place in a dimly lit and quiet room. Subjects were seated in front of a computer screen (placed at about 60 centimeters from subjects’ head) with headphones covering their ears. After a brief familiarization to the tasks, the different conditions were administered in pseudo-random order, counterbalanced between subjects and groups. In all conditions, subjects were instructed to press the left mouse button, as quickly as possible, at the target presentation. The targets were a small circle (unimodal visual conditions), the “high” or the “low” frequency sound (unimodal auditory conditions), the combination of a high frequency sound with a small circle or the combinations of a low frequency sound with a small circle (bimodal conditions). In the go/no-go conditions subjects were instructed not to answer to distractor stimuli. An illustration of the go/no-go conditions is displayed in Fig. 1.

### Data analyses

Data reduction was first applied to deal with errors and outliers in the response time data: (1) trials with incorrect responses were excluded from the response time analyses (3.78% of the trials) and (2) response times beyond 2.5 standard deviations below or above each participant’s mean for each experimental condition were discarded as outliers (0.45% of the trials). Analyses of variance (ANOVA with F-tests) were performed on the mean response times for the target detection and the percentages of false alarms in the no-go trials. Statistical analyses were performed using STATA/SE 12.0 for Windows (Stata Corp LP).

### Results

#### Clinical scores (subjects characteristics, questionnaires and scales)

There was no group difference for age [t(32) = 0.04, *p* = 0.48], educational level [t(32) = 0.92, *p* = 0.18], depression (BDI [t(32) = 0.03, *p* = 0.54], SDS [t(32) = 0.86, *p* = 0.20]), anxiety [t(32) = 1.63, *p* = 0.08] and hearing acuity (hearing loss) [t(32) = 1.34, *p* = 0.09] (see Table 2 for scores). THI and VAS scores were not correlated.

#### Psychophysical measurements

**Fig 2:**
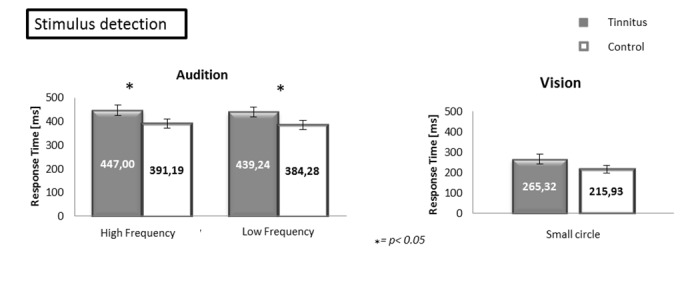
Response times during the stimulus detection conditions in the auditory (left) and visual (right) modalities. In the auditory modality, the response times are displayed as a function of the group and the frequency of the stimuli. In the visual modality, the response times are displayed as a function of the group. Error bars represent standard errors of the mean (SEM). (* p<0.05).

Stimulus detection: Two separate analyses of variance (ANOVA) were performed on the response times for stimulus detection: one in the auditory modality and one in the visual modality (Fig. 2). In the auditory modality, a 2 (group: TP vs CS) x 2 (frequency: tinnitus/high vs low)) ANOVA revealed an effect of the group [F(1.64) = 13.72, *p*<0.001], no effect of the frequency [F(1.64) = 0.24, *p* = 0.6253] and no interaction [F(1.64) = 0.01, *p* = 0.9775]. TP were slower than CS. In vision, a one-way ANOVA revealed no effect of the group [F(1.32) = 2.36, *p* = 0.1344] (see Fig. 2).

**Fig 3:**
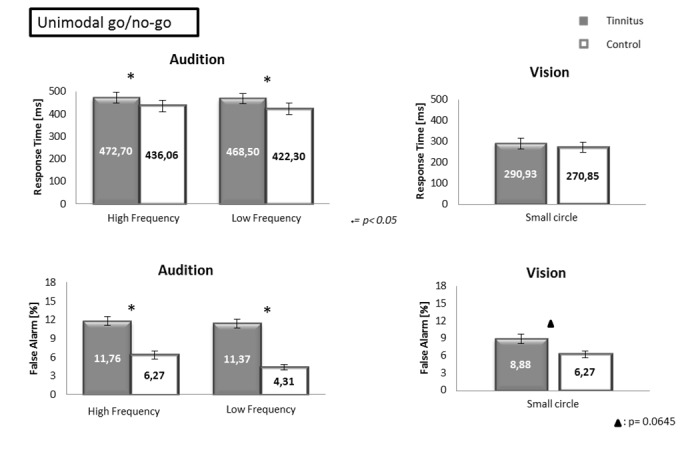
Response times and false alarm rates during the unimodal auditory and visual go/no-go tasks in tinnitus patients and control subjects. The upper part of the figure shows the response times for the unimodal go/no-go tasks in the auditory (left) and in the visual modality (right) as a function of the target. The lower part of the figure shows the percentage of false alarms for the same tasks as a function of the target. Error bars represent standard errors of the mean (SEM). * p<0.05; ▲trend p = 0.0645.

Unimodal go/no-go: Separate ANOVAs were performed within each sensory modality (Fig. 3). In the auditory modality, a 2 (group: TP vs CS) x 2 (frequency: tinnitus/high vs low) ANOVA performed on the response times showed an effect of the group [F(1.64) = 29.83, *p*<0.001], no effect of frequency [F(1.64) = 1.40, *p* = 0.2408] and no interaction [F(1.64) = 0.40, *p* = 0.5313]. The same ANOVA performed on the false alarm rates showed an effect of the group [F(1.64) = 12.45, *p*<0.001], no effect of the frequency [F(1.64) = 0.44, *p* = 0.5105] and no interaction [F(1.64) = 0.19, *p* = 0.6606]). TP had longer response times and made more false alarms than CS. In the visual modality, a one-way ANOVA performed on the response times showed no effect of the group [F(1.32) = 0.89, *p* = 0.3518]. The same ANOVA performed on the false alarm rates showed a trend towards a group difference [F(1.32) = 1.99, *p* = 0.0645].

**Fig 4:**
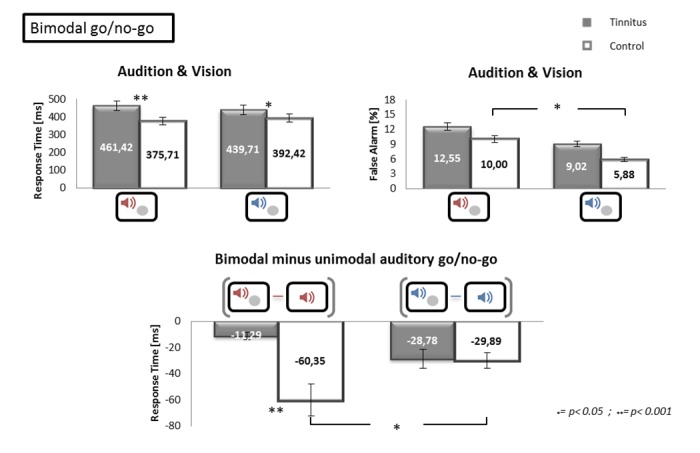
Results during Bimodal go/no-go tasks as a function of the group and the target. The upper part of the figure shows the response times (left) and the percentage of false alarms (right) for the bimodal go/no-go tasks as a function of the targets. In the lower part of the Figure, we show the results of the subtraction of the individual response times, i.e. bimodal minus unimodal auditory go/no-go. Error bars represent standard errors of the mean (SEM). * p<0.05; ** p<0.001.

Bimodal go/no-go: A 2 (group: TP vs CS) x 2 (frequency: tinnitus/high vs low) ANOVA performed on the response times showed an effect of the group [F(1.64) = 39.11, *p*<0.001], no effect of the frequency [F(1.64) = 0.06, *p* = 0.8152] and no interaction [F(1.64) = 3.26, *p* = 0.0756] (see Fig. 4). The same ANOVA performed on the false alarm rates revealed a trend towards an effect of the group [F(1.64) = 3.88, *p* = 0.0531], an effect of the frequency [F(1.64) = 7.03, *p*<0.05] and no interaction [F(1.64) = 0.04, *p* = 0.8387]. To compare bimodal to unimodal conditions in the auditory modality, we subtracted the individual response times in auditory unimodal go/no-go conditions from the individual response times in bimodal conditions (Fig. 4). A 2 (group: TP vs CS) x 2 (frequency: tinnitus/high vs low) performed on these values showed an effect of the group [F(1.64) = 5.85, *p*<0.05], no effect of the frequency [F(1.64) = 0.39, *p* = 0.5339], and an interaction effect [F(1.64) = 5.35, *p*<0.05]. Post-hoc comparisons using a Wald’s test with Bonferroni’s corrections showed that the gain in response time in bimodal versus unimodal auditory go/no-go was significantly higher in CS than TP for the tinnitus/high frequency stimuli (*p*<0.001) and not for the low frequency stimuli. Indeed, the gain in response time in bimodal versus unimodal auditory go/no-go was significantly higher for the tinnitus/high frequency stimuli than for the low frequency stimuli in CS (*p*<0.05). The correlations between the RTs and false alarm rates with the THI score and score of annoyance were not significant in TP (all p values >0.05).

### Discussion of experiment 1

In the unimodal conditions, TP had longer response times than CS during stimulus detection and unimodal go/no-go in the auditory modality only. In addition, TP made more false alarms than CS in both modalities (this was significant in the auditory modality and there was a trend in the visual modality). This indicates that the inhibitory control is altered in TP. In the bimodal conditions, TP had longer response times than CS in all conditions, especially when the tinnitus/high frequency sound was used as target. In addition, when comparing bimodal with unimodal auditory go/no-go conditions, we observed that TP did not show the same improvement (i.e. decrease) in response times than CS when tinnitus-matched high frequency sounds were used as targets. This indicates that the perception of high frequency sounds close to tinnitus frequency in TP interferes with sensory processing, precluding the normally expected improvement in bimodal conditions. This provides a convincing evidence of the strong interference power of sounds resembling to tinnitus, hypothetically because of an automatic and interfering emotional response in link with tinnitus.

## Experiment 2

In this second experiment, we used a unimodal go/no-go paradigm with a pre-stimulus in the untested modality to assess the sensitivity to cross-modal interference in TP and CS during auditory and visual go/no-go tasks. We also tested the effect of the sound frequency used for the target and pre-stimulus on performance, comparing tinnitus-matched/high frequency versus low frequency sounds.

### Materials and methods

**Fig 5:**
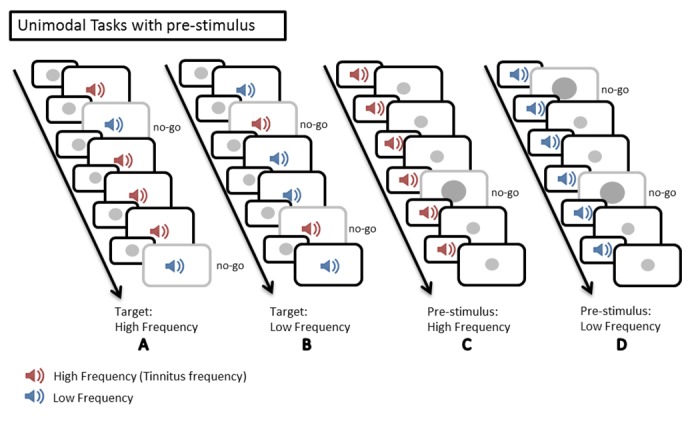
Illustration of unimodal go/no-go tasks with pre-stimulus. (A) An unimodal auditory task with visual pre-stimulus. Participants were instructed to press a mouse button at the presentation of the target (tinnitus/high frequency sound (in red)) and not at the presentation of the distractor (a low frequency sound (in blue)). (B) An unimodal auditory task with visual pre-stimulus. Participants were instructed to press a mouse button at the presentation of the target (a low frequency sound (in blue)) and not at the presentation of the distractor (a tinnitus/high frequency sound (in red). In A and B, before each stimulus (target or distractor) visual pre-stimulus (small circle) appeared. (C) and (D) Unimodal visual tasks with auditory pre-stimuli. Participants were instructed to press a mouse button at the presentation of the target (the small circle) and not at the presentation of the larger circle (distractor). The pre-stimulus was a tinnitus/high frequency or a low frequency sound respectively.

The same participants as in Experiment 1 took part to the Experiment 2. The same unimodal auditory and visual go/no-go conditions as in Experiment 1 (stimuli and tasks) were also used, except that the presentation of each stimulus was preceded either 150, 250 or 350 ms before by a pre-stimulus appearing in the untested modality (e.g. sound in the visual go/no-go) (Fig. 5). Four conditions were assessed: tinnitus/high frequency sound as the target preceded by a small circle, low frequency sound as the target preceded by a small circle, small circle as the target preceded by a tinnitus/high frequency sound as the pre-stimulus and small circle as the target preceded by a low frequency sound as the pre-stimulus.

### Data analyses

Data reduction was first applied to deal with errors and outliers in the response time data and analyses of variance (ANOVA with F-tests) were performed on the mean response times for the target detection and the percentages of false alarms similarly as in experiment 1.

### Results

**Fig 6:**
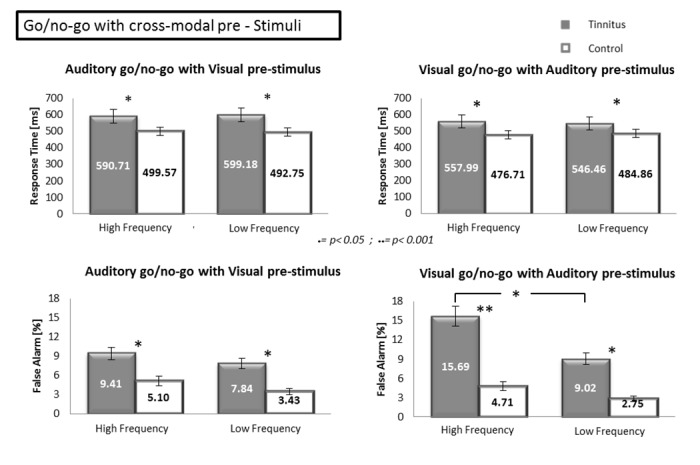
Response times and false alarm rates during the unimodal auditory and visual go/no-go tasks with pre-stimulus in tinnitus patients and control subjects. The upper part of the Figure shows the response times for the unimodal go/no-go tasks with pre-stimulus in the auditory modality (left) as a function of the target and in the visual modality (right) as a function of the pre-stimulus. The lower part of the figure shows the percentage of false alarms for the same tasks. Error bars represent standard errors of the mean (SEM). * p<0.05; ** p<0.001.

Separate ANOVAs were performed for auditory and visual targets. In the auditory go/no-go with visual pre-stimulus, a 2 group (TP vs CS) x 2 (target frequency: tinnitus/high vs low) ANOVA was performed on the response times and showed an effect of the group [F(1.64) = 14.42, *p*<0.001], no effect of the frequency [F(1.64) = 0.01, *p* = 0.9747] and no interaction [F(1.64) = 0.09, *p* = 0.7698] (Fig. 6). The same ANOVA performed on the false alarm rates showed an effect of the group [F(1.64) = 7.68, *p*<0.05], no effect of the frequency [F(1.64) = 1.05, *p* = 0.3082] and no interaction [F(1.64) = 0.01, *p* = 0.9755]). In the visual go/no-go with auditory pre-stimuli, a 2 (group: TP vs CS) x 2 (pre-stimulus frequency: tinnitus/high vs low) ANOVA performed on the response times showed an effect of the group [F(1.64) = 12.03, *p*<0.001], no effect of the frequency [F(1.64) = 0.01, *p* = 0.9347] and no interaction [F(1.64) = 0.23, *p*<0.6343]). A same ANOVA performed on the false alarm rates revealed a major effect of the group [F(1.64) = 32.88, *p*<0.001], a small effect of the frequency [F(1.64) = 8.22, *p*<0.05] and no interaction [F(1.64) = 2.45, *p* = 0.1227]. The correlations between the RTs and false alarm rates with the THI score and score of annoyance were not significant in TP (all p values>0.05).

**Fig 7:**
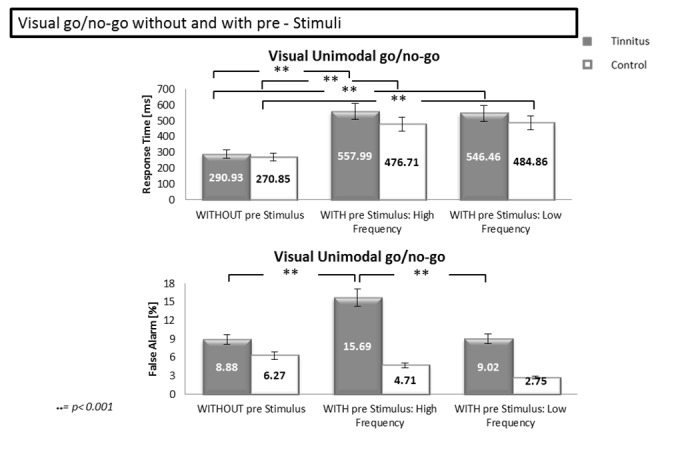
Response times and false alarm rates during the visual go/no-go without and with pre-stimuli. The upper part of the Figure shows the response times for visual unimodal go/no-go tasks without auditory pre-stimulus and with pre-stimulus of high and low frequency. The lower part of the Figure shows the percentage of false alarms for the same tasks. Error bars represent standard errors of the mean (SEM). * p<0.05; ** p<0.001.

To further analyze the effect of the auditory pre-stimulus frequency on performance in the visual go/no-go, we compared the results from the “classical” unimodal go/no-go (Experiment 1) with those from the go/no-go with pre-stimuli (Experiment 2). A 2 (Group: TP vs CS) x 3 (pre-stimulus: none, tinnitus/high frequency, low frequency) ANOVA with repeated measures was performed on the response times and showed a group effect [F(1.32) = 5.30, *p*<0.05], an effect of pre-stimulus [F(1.32) = 9.36, *p*<0.001] and an interaction [F(1.32) = 4.09, *p*<0.05] (Fig. 7). Post-hoc analyses revealed a significant difference between conditions (with and without pre-stimulus) in the tinnitus group, with shorter response times in the visual go/no-go without pre-stimulus than with high frequency pre-stimulus (p<0.001) and with low frequency pre-stimulus (p<0.001). The same effect was observed in the control group (i.e. significant shorter response times in the visual go/no-go without pre-stimulus than with pre-stimuli: high frequency (p<0.001) and low frequency (p<0.001)). The same ANOVA was performed on the false alarm rate and showed a group effect [F(1.32) = 23.48, *p*<0.001], an effect of the pre-stimulus [F(1.32) = 5.50, *p*<0.05] and an interaction [F(1.32) = 5.13, *p*<0.05]. Post-hoc analyses revealed a significant difference between conditions in the tinnitus group, with more false alarms in the visual go/no-go with tinnitus/high frequency pre-stimulus than in the two other conditions: low frequency pre-stimulus (p<0.001) and without pre-stimulus (p<0.001).

**Fig 8:**
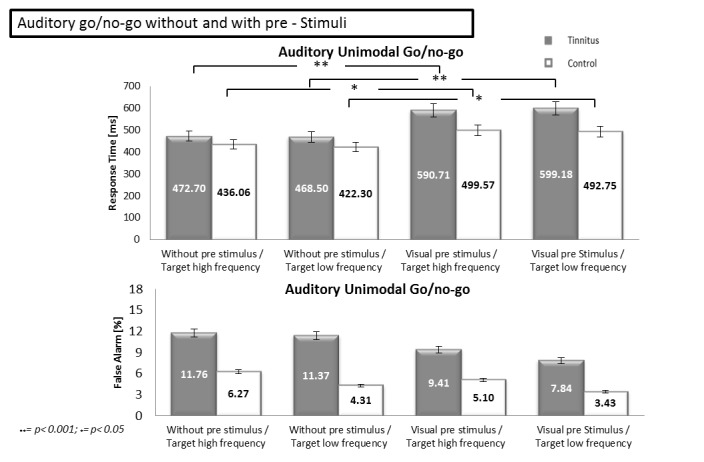
Response times and false alarm rates during the auditory go/no-go without and with pre-stimuli. The upper part of the Figure shows the response times for auditory unimodal go/no-go tasks without pre-stimulus and with visual pre-stimuli as a function of the auditory target. The lower part of the figure shows the percentage of false alarms for the same tasks. Error bars represent standard errors of the mean (SEM). * p<0.05; ** p<0.001.

To analyse the effect of a visual pre-stimulus on the performance in the auditory go/no-go, we compared the results from the “classical” unimodal go/no-go for the high and low frequency target (Experiment 1) with those from the corresponding go/no-go with visual pre-stimuli (Experiment 2). A 2 (Group: TP vs CS) x 2 (Target frequency: high frequency vs low frequency) x 2 (Pre-stimulus: none vs visual) ANOVA was performed on the response times and showed an effect of the group [F(1.128) = 26.77, p<0.001], no effect of the target frequency [F(1.128) = 0.09, p = 0.764] and an effect of the pre-stimulus [F(1.128) = 49.85, p<0.001] (Fig. 8). There was an interaction between the group and the pre-stimulus [F(1.128) = 4.48, p<0.05]. No other interaction was observed: interaction between the group and the target frequency [F(1.128) = 0.21, p = 0.647], interaction between the target frequency and the pre-stimulus [F(1.128) = 0.13, p = 0.718], interaction between the group, the target frequency and the pre-stimulus [F(1.128) = 0.01, p = 0.916]). Post-hoc analyses showed a difference between the conditions with and without visual pre-stimuli (pre-stimulus conditions) in both tinnitus (p<0.001) and control (p<0.05) groups. In the two groups, we observed shorter response times in the auditory go/no-go without pre-stimulus than with pre-stimuli. The same ANOVA was performed on the false alarm rate and showed an effect of the group [F(1.128) = 20.05, p<0.001], no effect of the target frequency [F(1.128) = 1.38, p = 0.242] and no effect of the pre-stimulus [F(1.128) = 2.79, p = 0.097] (Fig. 8). There was no interaction between the factors: interaction between the group and the target frequency [F(1.128) = 0.12, p = 0.726], interaction between the group and the pre-stimulus [F(1.128) = 0.64, p = 0.422], interaction between the target frequency and the pre-stimulus [F(1.128) = 0.03, p = 0.853], interaction between the group, the target frequency and the pre-stimulus [F(1.128) = 0.09, p = 0.757]).

### Discussion of Experiment 2

In all the go/no-go conditions with pre-stimuli, TP had longer response times and made more false alarms than CS. The strongest interference effect was observed in TP during the visual go/no-go with tinnitus/high frequency pre-stimulus, while similar effect of the pre-stimuli was observed in the other conditions and in particular in TP. The present study brings evidences that TP are abnormally sensitive to cross-modal interference, affecting the inhibitory control, particularly when sounds resembling to tinnitus perception are present.

## General Discussion

Until now, very few studies have investigated the nature of the cognitive impairments in tinnitus patients (TP). The purposes of the present study were to test the efficiency of inhibitory control mechanisms in the auditory and visual modalities as well as the sensitivity to cross-modal interference. In the present study, we showed that TP had slowed down response speed during stimulus detection in the auditory modality. Throughout Experiments 1 and 2, results consistently indicated that TP were less accurate and slower than control subjects (CS) during both the auditory and the visual go/no-go conditions. Finally, TP were more sensitive to cross-modal interference than CS, in particular when pre-stimuli resembling to the tinnitus perception were used even if the pre-stimuli were not relevant for the task.

### Slowed down reaction speed in tinnitus

The longer response times observed in TP during the auditory stimulus detection tasks suggest that tinnitus affects sensory processing mechanisms in the auditory modality as previously proposed by Das and colleagues [30]. One may hypothesize that the constant perception of phantom sensations (tinnitus) would engage parts of the auditory processing system, making it slower to react to other (external) sound stimulations. This phenomenon may involve the so-called irrelevant sound effect: the presence of irrelevant (low intensity) sounds affects performances in various cognitive tasks [31]. In the case of tinnitus, in addition to the interference in working memory, the phantom auditory perceptions are in competition with the processing of external sounds and monopolize part of the auditory system explaining why TP are more affected in the auditory modality.

### Altered inhibitory control in tinnitus

TP were less accurate and slower than CS during unimodal go/no-go tasks, in particular during auditory conditions. In bimodal conditions, TP were slower than CS and there was an effect of the frequency on the response accuracy. These results in the different go/no-go tasks clearly indicate that the inhibitory control mechanisms are impaired in tinnitus. This impairment may be related to other alterations previously reported in tinnitus, such as impairment in selective attention [21–22]. In selective attention tasks, the cognitive control and, in particular, the inhibitory control plays a crucial role in the suppression of irrelevant information [32–36]. We postulate that alterations in these cognitive control mechanisms would play a key-role in tinnitus generation and maintenance. The alteration of the cognitive control could be due to the occurrence of negative emotional responses caused by the perception of a stimulus with an emotional meaning (e.g. tinnitus), similarly to what has been described in pain perception [37–38]. This hypothesis is consistent with recent models that postulate a role of limbic structures in tinnitus [4, 6]. In accordance with this view, hyperactivity in limbic structures (e.g. the nucleus accumbens) has been reported in response to auditory stimulations in TP, in particular when tinnitus-matched frequency sounds were used [7]. Auditory stimulation in TP also elicits brain activity in the distress network, i.e. the anterior cingulate cortex, anterior insula, and amygdala [6, 39].

When comparing bimodal and auditory unimodal tasks in regard to the frequency used, results indicated shorter response times in CS when the “go” stimulus was the combination of a high frequency sound and a small circle. This combination corresponds to a “natural” crossmodal correspondence previously described, which would explain the optimal performance in CS for this combination [40–42]. Surprisingly, this effect was not observed in TP. The expected improvement in the response times for the bimodal conditions when compared to the unimodal auditory go/no-go was even smaller for the “optimal combination” than the one observed for the other combination (low frequency and the small circle). We hypothesize that the interference produced by the presentation of a high frequency stimulus that resembled to tinnitus perception was stronger than the facilitating effect induced by the presentation of the “optimal combination”.

From a neural point of view, go/no-go tasks are usually associated with the inhibitory control [16] and involve the ventrolateral prefrontal cortex (vlPFC) and dorsolateral prefrontal cortex (dlPFC). The dlPFC is also known to exert early inhibitory modulation of input to primary auditory cortex in humans [43], which is thought to be compromised in TP [44]. Noteworthy, the stimulation of the dlPFC using transcranial direct current stimulation (tDCS) or transcranial magnetic stimulation (TMS) affects both tinnitus intensity and distress [44–46]. The vlPFC is described as part of the network required to generate conscious perception of tinnitus [8] and its stimulation using TMS modulates the loudness of tinnitus perception [47]. Additionally, these areas (dlPFC and vlPFC) are involved in the regulation of emotions [48–49]. In tinnitus, the cognitive and emotional aspects are probably intimately interconnected and influence each other, so that the alteration of one component affects the functioning of the other one, resulting in tinnitus perception. In accordance, the ventromedial prefrontal cortex (vmPFC) that is altered in tinnitus [7, 50–51] is connected to the vlPFC and involved in the affective valuations of the stimulus [49].

### Increased cross-modal interference in tinnitus

The presence of a pre-stimulus affected the performances in both groups when compared to the “classical” unimodal go/no-go conditions. However, the effect was stronger in TP, in particular when high frequency sounds resembling to tinnitus perception were used as pre-stimuli. This indicates that TP are abnormally sensitive to cross-modal interference. The strongest effect caused by the tinnitus-like pre-stimuli may be due to the occurrence of an automatic emotional response in link with tinnitus that would briefly disturb the cognitive functioning (inhibitory control). It has been shown that suppressing an emotionally significant sound affects performances in cognitive tasks [52].

These interferences from sensory stimulation and/or tinnitus perception are normally regulated by the cognitive control that is impaired in TP and that prevent unwanted stimuli and/or memories to reach consciousness [53–54]. Noteworthy, part of the neural network that plays a role in the inhibitory control (go/no-go tasks) [17, 55–56] and information filtering [33, 57–58] is also involved in tinnitus perception [8]. In addition, the auditory efferent system [59] could be altered in tinnitus and contribute to some extent to the strongest sensitivity to cross-modal interference in TP [60]. In sensory selective attention tasks, the efferent system is supposed to reduce the attention toward irrelevant stimuli in the other sensory modalities [61–62]. Alterations in the efferent system may also contribute to the tinnitus perception [63].

In conclusion, we showed here that tinnitus is associated with an impairment of the inhibitory control, as well as a high sensitivity to the sensory interference, especially when stimulations resembling to the tinnitus perception are used. We postulate that alterations in these cognitive control mechanisms would play a key-role in tinnitus generation and maintenance. Future studies using functional brain imaging should further establish a direct link between inhibitory control and alterations of the dorsolateral and ventrolateral prefrontal cortex in tinnitus patients.
